# Brief report of a pilot test of an audit tool for assurance of Declarations of Interest policy in NICE COVID‐19 guidance production

**DOI:** 10.1002/gin2.70007

**Published:** 2025-04-09

**Authors:** Harriet Edmondson, Hannah Patrick, Sarah Boyce, Margaret McCartney, Anneka Patel, Maria Majeed, Susan Bewley, Kevin Harris

**Affiliations:** ^1^ Public Health Registrar (ST5) East of England Deanery Cambridge United Kingdom; ^2^ National Institute for Health and Care Excellence NICE London United Kingdom; ^3^ School of Medicine University of St Andrews St Andrew's Scotland United Kingdom; ^4^ Department of Women's and Children's Health King's College London London United Kingdom; ^5^ College of Life Sciences University of Leicester Leicester United Kingdom

**Keywords:** audit and feedback, clinical practice guideline, conflicts of interests' disclosure and handling, guidelines methodological topics and challenges, implementation and communication topics, type of guideline submitted

## Abstract

**Introduction:**

The National Institute for Health and Care Excellence (NICE) UK helps practitioners and commissioners to achieve high‐quality, cost‐effective patient care by publishing relevant evidence‐based recommendations, including guidelines. These are developed by NICE advisory committees comprising health and care professionals, service users, and their carers. To ensure NICE's independence it has a comprehensive policy on declaring and managing interests for advisory committees. The application of the policy is not straightforward and the aim of this work was to pilot test an audit tool for external assurance of self‐declared interests; and to produce methodology with wider applicability for other research and guideline‐producing organisations.

**Methods:**

A pilot study of adherence to the NICE DOI policy was undertaken. The number and type of DOIs made by the 48 panel members developing the NG191 Managing COVID‐19 guideline was assessed, using multiple external sources to check for undeclared interests. DOIs were reviewed from a 6‐month period at the height of activity (1 March–31 August 2021). DOIs were checked by assessors (experienced NICE staff involved in application of the DOI policy) against a comprehensive search strategy including multiple public sources (PubMed, Google Scholar, Disclosure, etc.) of information for undisclosed DOIs over the 12 months preceding the member's contribution and the 12 months during which they contributed.

**Results:**

There was a total of 126 unique self‐declared interests during the 6‐month time period and we found a further 280 undeclared interests. Of these 280, 75 were deemed likely to be relevant (27%) but only 10 (4%), would have led to a request for more information from the panel member had it been declared (according to DOI policy version 1.4 in place at that time). The team considered that 7 of the undeclared interests would have led to exclusion from decision‐making had they been known (3% of the undeclared interests). All related to academic output.

**Discussion:**

This pilot provided useful insight into NICE DOI policy compliance. The methodology needs further testing and development for wider, routine contexts. A digital version of the audit tool would enable routine, more efficient audit of declarations of interest. This study was an important quality assurance exercise from which we have been able to propose methodology that can be automated and widely adopted.

## Key points

*Key findings*

- Our audit identified a significant number of undeclared interests but a small proportion of those would have required further investigation and only 3% had implications for involvement in decision making.
*What this adds to what is known now*
- Organisations that produce and publish guidelines, or research need to manage potential Conflicts Of Interest (COI) to avoid bias. The application of policies relating to Declarations of Interest is not straightforward and can be inconsistent. There has been increasing interest among academics and the public around COIs in regulatory and guidance bodies.
*What is the implication and what should change now*
- The NICE DOI has subsequently been updated for to clarify which interests should be declared and the organisation is developing an automated tool to enable routine audit of declarations.

## 1 INTRODUCTION

The National Institute for Health and Care Excellence (NICE) UK helps practitioners and commissioners to achieve high‐quality, cost‐effective patient care by publishing relevant evidence‐based recommendations, including guidelines. NICE has advisory committees comprising health and care professionals, service users, and their carers to draft recommendations. To ensure NICE's independence it has a comprehensive policy on declaring and managing interests for advisory committees.^1^ NICE's definition of a conflict of interest (COI) is ‘a set of circumstances that creates a risk that professional judgement or actions regarding a primary interest will be unduly influenced by a secondary interest’.^2^ The policy provides guidance on ‘when a declared interest could represent a COI and the action that should be taken to manage this (ranging from simple open declaration to complete exclusion from NICE committee work)’.

As with other organisations managing COIs, including those that publish guidelines, or research, the application of policies is not straightforward and can be inconsistent.^3^, ^4^ There has been increasing interest amongst academics and the public around COIs in regulatory and guidance bodies, including at NICE.^5^, ^6^, ^7^

The NICE policy relies on self‐declaration of panel members' interests. Validation against external sources demonstrates that variation in interpretation is possible. NICE's COVID‐19 guidance production was faster than historically had been the case and required real‐time ongoing revision as evidence developed. This brought some unique challenges, to NICE's existing DOI policy which remained unchanged and NICE staff were keen to check that the DOI policy had been appropriately applied.

The aim of this work was to pilot test an audit tool for external assurance of self‐declared interests in the context of rapid guidance development; and to produce methodology with wider applicability for other research and guideline‐producing organisations.

A pilot study of adherence to the NICE DOI policy was undertaken. The number and type of DOIs made by the 48 panel members developing the NG191 Managing COVID‐19 guideline was assessed, using multiple external sources to check for undeclared interests. DOIs in the COVID‐19 guidance programme were reviewed from a 6‐month period at the height of activity (1 March–31 August 2021) (Table 1).

**Table 1:** Self‐declared interests (MAC registry) and interests found on full search strategy for 48‐panel members.

Source	Mean/median (range) of interests/panel member
MAC Registry self‐declared interests	3.8/3 (0–20)
PubMed	5.3/0 (0–58)
Google search 1	2.1/1 (0–12)
LinkedIn	1.3/0 (0–6)
NHS Trust site/GP surgery site/any private practice	0.9/0 (0–8)
Google search 2	0.6/0 (0–7)
Companies House	0.4/0 (0–4)
Disclosure UK (APBI)	0.1/0 (0–1)
ClinicalTrials	0.1/0 (0–1)
Sunshine UK	0.1/0 (0–2)
Private hospital chains	0.1/0 (0–2)
NICE/SIGN	0.1/0 (0–1)
Google Patents	0.1/0 (0–1)
Private practice website	0.03/0 (0–1)
Electoral Commission	0/0 (0)
OpenPayments	0/0 (0)
Drug Manufacturer website	0/0 (0)

Abbreviations: APBI, Association of British Pharmaceutical Industry; MAC, Managing Acute COVID‐19; SIGN, Scottish Intercollegiate Guidelines Network.

The following steps were taken to review and cross‐check the accuracy of DOIs:

- The DOI registry was reviewed for all interests declared and their management.
- DOIs were checked by assessors (experienced NICE staff involved in application of the DOI policy) against a comprehensive search strategy including multiple public sources (PubMed, Google Scholar, Disclosure, etc.) of information for undisclosed DOIs over the 12 months preceding the member's contribution and the 12 months during which they contributed.^8^ (Appendix A for full search strategy as advised by Dr. McCartney).
- Descriptive analysis was undertaken to identify the sources where undisclosed interests were most frequently uncovered during the full analysis; these were retained to develop a simplified search strategy (for wider future use as the full strategy proved too time‐consuming for routine application) (Appendix B). The simplified search strategy was used to validate findings from the first stage. Four members of the study team (authors of this paper) volunteered to repeat the audit for 12% of searches. The validation was undertaken by repeating the search originally performed by another team member. This identified 7 additional interests of which none were related to a problem with the search strategy itself, but rather interpretation or methodology used by the study volunteer.
- Undeclared interests found by using the full search strategy and any additional interests found via the simplified strategy were reviewed for their relevance by a Senior Technical Analyst and Programme Manager for the COVID‐19 team (AP, MM, SB) and then by the Consultant Clinical Advisor for the COVID‐19 team (KH). This process being exactly what is routinely done.

There was a total of 126 unique self‐declared interests in the MAC registry during the 6‐month time period and we found a further 280 undeclared interests. Of these 280, 75 were deemed likely to be relevant (27%) but only 10 (4%), would have led to a request for more information from the panel member had it been declared (according to DOI policy version 1.4 in place at that time). Table 2 presents information relating to the type (category) of 10 undeclared conflicts identified, 7 of which the team considered would have led to exclusion from decision‐making had they been known (3% of the undeclared interests). All related to academic output.

**Table 2:** Undeclared ‘possibly’ relevant interests by category and number that should have been investigated further (as assessed by experienced senior members of the NICE team).

Category	Number (%)	Conflict
Healthcare/pharmaceutical/industry company role or fees	4 (5%)	0
Investigator/author for relevant clinical study/academic work including studies used in the guideline, studies on the clinical topic of the guideline (e.g., therapeutic management of COVID‐19).	23 (31%)	10
Clinical and/or academic professional role aside from primary employment stated to NICE	20 (27%)	0
Healthcare operational, guideline or leadership role relevant to topic of guideline, that is, COVID‐19, acute care, etc.	23 (31%)	0
Private practice role in field relevant to the guideline, for example, respiratory medicine, radiology, neurology.	5 (7%)	0
Total	75	10

Undeclared interests were most often found via Pubmed (156/280, 56%), and Google searches (53/280, 19% first Google search; 11/280,4% second Google search).

Our pilot study confirmed that there is scope for improvement in NICE's policy of declaration and checking of interests, albeit the DOI policy is in line with and often stricter than other similar organisations (e.g., the International Committee of Medical Journal Editors statement on COIs is less proscriptive in its definition and management of relevant interests). As the initial strategy was time‐consuming, a simplified one was employed and found to be robust. The full strategy may need to be adapted differently by other NICE teams, depending on the area covered (particularly if involving private surgical practice).

This pilot provided useful insight into NICE DOI policy compliance. It was conducted in an atypical context of the COVID outbreak, and the methodology needs further testing and development for wider, routine contexts. Factors to consider would be other sources of possible information on relevant interests, particularly for interventional procedures and devices used in a private context. In addition, it would need to be adapted for use in differing national contexts, for example, using European equivalents to the Disclosure Database. The methodology was resource hungry, and a digital version of the audit tool would enable routine, more efficient audit of declarations of interest. The NICE Declaration of Interest policy (v1.4) has subsequently been amended to include a requirement to declare all potentially relevant paid employment to the committee (v1.5) and NICE is currently investigating how digital technologies might implement the simplified strategy in a streamlined proportionate use of administrative resource. This study was an important quality assurance exercise from which we have been able to propose methodology that can be automated and widely adopted.

## AUTHOR CONTRIBUTIONS

All authors have worked for or contributed to, the work of NICE on an advisory basis. As such we all have an interest in quality assurance of NICE output.

## CONFLICT OF INTEREST STATEMENT

The authors declare no conflict of interest.

## ETHICS STATEMENT

The work did not require patient consent, clinical trial registration or research ethics approval.

## Data Availability

The data that support the findings of this study are available on request from the corresponding author. The data are not publicly available due to privacy or ethical restrictions.

## APPENDIX A FULL SEARCH STRATEGY

This method of searching for interests was based on previous studies which used similar methodology.^9^ The search strategy employed has previously been tested but is not expected to be capable of finding all possible undeclared interests. It was developed for this study into an Excel tool.

Where not specified, use search function on website and try ‘firstname lastname’ and ‘lastname’. Interests from the 12 months preceding declaration are relevant (i.e., March 2020 at the earliest up to the point of submission).

1. Google search for job title, roles, affiliations—first five pages ‘Firstname Lastname’ + ‘NHS’ + ‘private’ + ‘clinic’.
2. Google: ‘Firstname lastname’ + ‘Conflicts of interest’ + ‘declared interests’.
3. Disclosure UK (APBI)—centralised disclosure database for the pharmaceutical industry: try ‘firstname lastname’ and ‘lastname’ and ‘lastname, city’: https://search.disclosureuk.org.uk/. Consider searching known companies of member.
4. NHS Trust site/GP surgery site/University/any private clinics—search for Register of Interests, and search within (if not publicly available consider need to write to NHS Trust using FOI if necessary): ‘firstname lastname’ and ‘lastname’.
5. NICE/SIGN search for (1) Firstname Lastname (2) if present, search for DOI.
6. PubMed Firstname Lastname—search for articles and declarations of interest: ‘firstname lastname’, if too many results ‘firstname lastname’ [author]. Can also apply date limits. If few results, alternatively/as well Google Scholar ‘firstname lastname’ or ‘intial lastname’.
7. Linkedin (search for proflile—‘firstname lastname’ or ‘initial lastname’, review summary page no need to review all posts.
8. Companies House: use search for a company tool: ‘firstname lastname’, first 20 results. Use location to work out correct person if lots of results.
9. Electoral Commission—personal or company donations: search the political finance database‐> donations and loans‐‐> ‘firstname lastname’ or ‘initial lastname’ and dates. earch.electoralcommission.org.uk.
10. Patents (Google Patents—patents.google.com): search with date range and ‘fisrtname lastname’ or ‘initial lastname’ in inventor and assignee boxes.
11. ClinicalTrials.gov: find a study:‐> ‘other terms’: ‘firstname lastname’ and ‘initial lastname’ THEN advanced search ‘firstname lastname’ and ‘initial lastname’ in ‘sponsor/collaborator’ and ‘sponsor (lead)’ with study start dates applies.
12. OpenPayments https://openpaymentsdata.cms.gov/: advanced search: provider firstname and provider lastname.
13. Sunshine UK (whopaysthisdoctor.org): search for a doctor ‘firstname lastname’ and ‘lastname’.
14. Searches of websites of manufacturers whose drugs were reviewed by the programme for funding disclosures: includes drugs that are newer and on‐patent. Relevant companies are: Gilead Sciences Ltd, Roche products ltd, Sanofi Genzyme.
15. Private practice websites of panel members.
16. The five largest private hospital chain websites (HCA, Spire, Circle Health Group (formerly BMI Healthcare), Ramsay, and Nuffield Health): search function or ‘search for a consultant’: ‘firstnamelalstname’ or ‘lastname’. https://www.spirehealthcare.com/sitemap/.
17. If results so far suggest activity abroad, review relevant DOI registry of that country (if it exists).

## APPENDIX B SIMPLIFIED SEARCH STRATEGY

Where not specified, use search function on website and try ‘firstname lastname’ and ‘lastname’.

Interests from the 12 months preceding declaration are relevant (i.e., March 2020 at the earliest up to the point of submission).

For doctors, it is worth checking the GMC register first which can give additional helpful information such as middle names, specialty, year of qualification to help ensure correct person is being found. https://www.gmc-uk.org/registration-and-licensing/the-medical-register.

1. General Google search for job title, roles, affiliations—first five pages ‘Firstname Lastname’ + ‘NHS’ + ‘private’ + ‘clinic’.
2. PubMed or Google scholar: search for articles and on each article check the declarations of interest: ‘firstname lastname’, if too many results ‘firstname lastname’ [author]. Suggest also apply date limits.
3. Linkedin (search for proflile—‘firstname lastname’ or ‘initial lastname’, review summary page (experience, education, activity) no need to review all posts.
4. Google: ‘Firstname lastname’ + ‘Conflicts of interest’ + ‘declared interests’.
5. NHS Trust site/GP surgery site/University/any known private practice (retrieved from google searches)—search for Register of Interests, and search within the site using the search function: ‘firstname lastname’ and ‘lastname’.
6. Companies House: ‘get information about a company tool’: ‘firstname lastname’, first 20 results. If lots of results: use location (place of work) to try to assess if correct person, exclude obviously irrelevant results, for example, for very unrelated types of work. https://www.gov.uk/get-information-about-a-company.
7. Disclosure UK (APBI)—centralised disclosure database for the pharmaceutical industry: try ‘firstname lastname’ and ‘lastname’ and ‘lastname, city’: https://search.disclosureuk.org.uk/. Consider searching known companies of member.
8. ClinicalTrials.gov: find a study:‐> ‘other terms’: ‘firstname lastname’ and ‘initial lastname’ THEN advanced search ‘firstname lastname’ and ‘initial lastname’ in ‘sponsor/collaborator’ and ‘sponsor (lead)’ with study start dates applies.
9. Patents (Google Patents—patents.google.com): search with date range and ‘fisrtname lastname’ or ‘initial lastname’ in inventor and assignee boxes.
10. Sunshine UK (whopaysthisdoctor.org): search for a doctor ‘firstname lastname’ and ‘lastname’.
11. If clinician in relevant specialty: five largest private hospital chain websites (HCA, Spire, Circle Health Group (formerly BMI Healthcare), Ramsay, and Nuffield Health): search function or ‘search for a consultant’: ‘firstnamelalstname’ or ‘lastname’.
